# New Directions in Bioinformatics

**DOI:** 10.6028/jres.094.008

**Published:** 1989

**Authors:** Daniel R. Masys

**Affiliations:** Lister Hill National Center for Biomedical Communications National Library of Medicine Bethesda, MD 20894

**Keywords:** biotechnology, computer communication networks, computer systems, database management systems, medical informatics, molecular biology, National Library of Medicine (U.S.)

## Abstract

Two decades have passed since the first large scale, public access computer-based information systems were developed to store and disseminate the knowledge of medicine and biology. These first systems were bibliographic, and though the searching of computer files of citations remains the most common use of biological databases, there are dramatic forces at work in basic biology which are driving a transition from the printed page to the factual database. Unlike bibliographic systems, which contain only a pointer to information located elsewhere, factual data-bases contain the information sought. Development of automated methods to sequence DNA, RNA, proteins, and other macromolecules have yielded oceans of cryptic symbols, for which there is an absolute dependence upon computerized factual databases to acquire, store, retrieve, and analyze data. The Human Genome Project has focussed attention on the information science aspects of nucleic acid data, yet for the practicing scientist nucleic acids and other sequence data are just one piece of an increasingly complex biological puzzle whose solution will be expressed in terms of structure and function. Access to and integration of information across multiple related biological data-bases is a major challenge facing information system builders, a challenge which holds the promise of creating knowledge synergy from what are today disconnected, stand-alone information sources.

## Background

The methods for acquiring, storing, manipulating, and disseminating scientific knowledge are changing rapidly. Though the printed page remains the principal medium for the publication of research results, in data intensive applications such as crystallography, the advantages of computers as generalized symbol processors are leading to a paradigm shift from the printed page to the factual database. Among the public institutions most profoundly affected by this change is the National Library of Medicine (NLM). NLM celebrated its 150th anniversary in 1986, honoring a long tradition as the world’s largest repository and distribution center for the published knowledge of medicine and biology. In 1971, the Library initiated the MEDLARS online bibliographic files including MEDLINE and now over 20 other databases. MEDLINE remains the most widely used bibliographic file in biomedicine, answering some 4 million online inquiries this past year.

The Lister Hill National Center for Biomedical Communications is the Research and Development Division of the Library, serving as an intramural laboratory analogous to the intramural labs of the other institutes of the NIH. The Lister Hill Center has a staff of about 80, many of whose professional backgrounds are a hybrid of biological or medical science and computer and information science. The Lister Hill Center conducts research and development in three major areas: Computer and Information Science as applied to biomedical research and health care delivery; Electronic Image technologies such as image capture, compression, storage on optical disc, retrieval and transmission; and Health Professions Education using new information technologies such as microcomputers and videodiscs.

## Biotechnology Information

A new era in the mission of the National Library of Medicine was inaugurated with the appointment of Dr. Donald Lindberg as its Director in 1984. One of his first strategic moves was, along with the Library’s Board of Regents, to commission a long range plan for the Library. Calling together some 120 experts in the fields of library science, computer and information science, and health professions education, he charged the Long Range Planning panels with picturing the future 5, 10, and 20 years hence, and made recommendations to move the Library into that information technology-rich future. Of the more than 80 recommendations made to the Library, that given the highest institutional priority was the development of better information systems in what has come to be known as Biotechnology [1].

Biotechnology information refers to the computerized factual databases of molecular and structural biology which are growing rapidly in size and number. In the setting of the growing Human Genome Project, the current central focus of these research databases is DNA in partucular, and sequence databanks in general. The development of automated methods to analyze DNA, RNA, and protein sequences has, for the first time in the history of biology, brought to a large fraction of scientists an absolute dependence upon computers for the representation, storage, retrieval, and analysis of this data which far exceeds the limits of human cognition to remember, to scan, and to detect patterns of significance.

**Figure 1:**
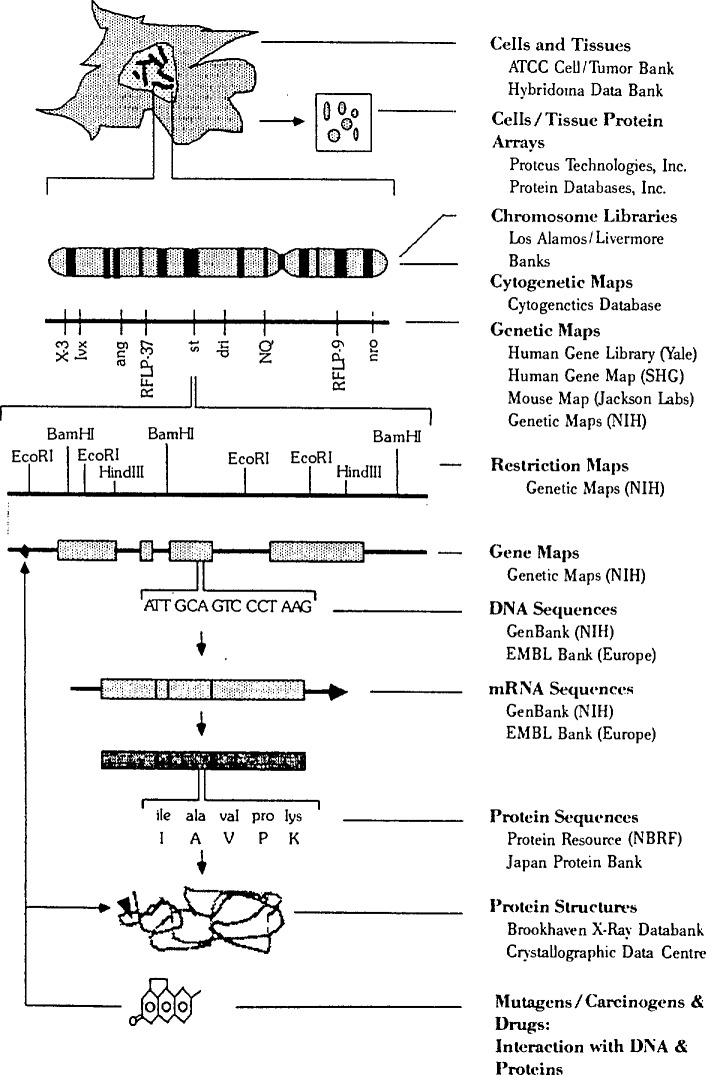
Biology knowledge bases.

There are a number of conceptually related but technically dissimilar databases which are national resources for molecular biology research, and most are supported by the NIH in one form or another (fig. 1). In the aggregate, they might be viewed as an electronic Tower of Babel, a maze of contrasting record structures and searching dialects which frustrates all but the most ardent researcher who wishes to access more than one of them. During the past 2 years, NLM has listened to the scientists, looked at its own institutional strengths, and has begun the work of devising better information management systems and methods for the knowledge of molecular and structural biology.

## Current NLM Biotechnology Activities

The NLM’s current activities fall into three major areas: the building and maintenance of data-bases relevant to molecular biology; new methods of retrieval and analysis of the information in those databases; and education, both for the researchers who generate the data and need to familiarize themselves with the computer-based tools being developed to facilitate management and analysis of the information, and for computer scientists who need to understand the data analysis problems of biologists.

In the area of database building and maintenance, several new computer index terms have been created which denote that an article contains nucleic acid or protein sequence information. The indexing coverage of the NLM will provide surveillance of nearly 3000 journals for the appearance of new literature in this area. As indexers apply the new tags to the literature, MEDLINE subsets will be made available in computer readable form to molecular biology database builders, to use either as a current awareness service for papers appearing in journals that they do not normally scan, or even as the kernel for new entries in their databases. Virtually every one of the factual databases of molecular biology has a bibliographic citation as a component of its unit record.

The MEDLINE searching vocabulary and computer record structures are being modified to enable automated linkage between the published literature and the DNA research databases. Once these links are in place, it will be possible for an investigator who is browsing through the DNA database to tell the computer to find literature related to one or a group of GenBank records. Going the other way, the computer will allow those who are doing literature searches in molecular biology to automatically connect to the databases where the actual data cited in the publication is contained. Although the DNA databases are the first proto-type linkage, we hope to extend this interlinkage to all of the major biology databanks, including biophysical and structural databanks such as Brookhaven and Cambridge.

Other new database building efforts include a database of databases, that is, a Directory of Biotechnology Information Resources. This collection will become an online file, so that if an investigator wants to know where a particular biotechnology database is located, and how to connect to it, he can browse our online catalog of research data-bases.

One important social and political aspect of biotechnology is the issue of environmental release of genetically altered organisms. At the request of the U.S. Biotechnology Science Coordinating Committee, the NLM is working with representatives from domestic U.S. agencies such as the EPA, Dept. of Agriculture, and NSF, and foreign delegates from the Commission of European Communities, and various European scientific databases to develop an environmental release database which will serve as both a scientific and regulatory information resource.

Since 1982, the Lister Hill Center has been supporting the development of Victor McKusick’s “Mendelian Inheritance in Man,” the acknowledged world standard reference describing over 4000 human genetic diseases. We have converted the entire textbook into a full text, online database, searchable by natural language query. Current efforts underway are enhancing the text with a library of images, including x rays and clinical photos of patients with the genetic diseases described in the text.

The building of new and more capable electronic systems for collecting and storing biotechnology information is not enough in itself to ensure progress. We need better ways of retrieving and analyzing that information, in a manner that is easy for both expert and novice users. The concept of simultaneous retrieval of related information from multiple databases is central to development work now underway in the Lister Hill Center’s Information Technology Branch. Our Information Retrieval Experiment, or IRX software, is being adapted to the special needs of biotechnology data-bases.

The system model is based on a windowing scientific workstation which allows natural language questions to be asked, with retrieval of related information from a number of conceptually related databases. The prototype system has simultaneous access to 12 different databases, including the Brookhaven crystal structures. New user interfaces based on high resolution windowing workstations, are a part of this work. A pseudo-natural language allows the user to state a question in his own language, such as “Has Factor XII deficiency been mapped?” The keywords of the query are parsed and truncated to form word stems, then all occurrences of those word stems are statistically ranked to generate an ordered retrieval set.

The new and rapidly changing knowledge of molecular and structural biology confers an ongoing need for education, and the Library has instituted a series of biotechnology lectures. These introductory lectures on the topics of the new genetics and the information science aspects of the field have been put on videotape and are available as part of NLM’s lending collection. Educational efforts in computer and information science as applied to molecular biology need also to be directed to the workers in the field. NLM has hosted more than 60 NIH molecular biologists for workshops on computerized “Molecular Sequence Analysis”.

## The Future in Computational Biology

It is clear that molecular and structural biology is an area of science which, perhaps more than any other component of biology or medicine, has acquired an absolute dependence upon computers to carry forward the advancement of science. At the NLM, the first major objective of the future will be building, maintaining, and providing access to research information resources, in much the same way that the Library organizes, indexes, and provides access to the scientific literature. The second objective will be support for scientific discovery.

Information Resources programs will include ongoing support for key molecular databases. NLM will continue to be one of many distribution channels for this kind of research information, using the Library’s extensive international MEDLARS network, and importantly, NLM will focus at the interfaces between these biology knowledge sources, and help develop and promote standards which will permit them to exchange data and provide access to conceptually related information located in multiple databases.

It is clear that the information resources will continue to evolve optimally only if they follow a vanguard of informatics research (i.e., scientists, who use the databanks as the substrate for experimentation and hypothesis testing and who can paint the scientific concepts which data structures must accomodate, must actively collaborate with those responsible for the development and application of the information resources). To this end, a focus of the Biotechnology Information Center Program is a core intramural research staff of hybrid computer scientists and biological scientists, complemented, as it is in the other intramural laboratories of the NIH, by a visiting scientist program. Grant support for the computational aspects of molecular and structural biology would be a substantial component of the program, as would the convening of tutorials, workshops, and scientific meetings highlighting the union of computer science and biological science.

These are exhilarating times in biology and also in information systems development; the National Library of Medicine will be pleased to work with each of the laboratories and institutions represented at this conference to improve the information tools of basic biology and their inevitable effect on the future practice of molecular medicine.
